# Semi-automatic 3D-quantification of in-vivo synapse formation

**DOI:** 10.1186/s12859-026-06480-6

**Published:** 2026-06-05

**Authors:** Blaž Brence, Laura R. Wandelt, Sophie Walter, Stephan J. Sigrist, Astrid G. Petzoldt, Daniel Baum

**Affiliations:** 1 Department of Visual and Data-Centric Computing, Zuse Institute Berlin, Takustr. 7, 14195 Berlin, Germany; 2 Institute of Biology, Freie Universität Berlin, Takustr. 6, 14195 Berlin, Germany

**Keywords:** Liprin-*α*, Synapse formation, Neuromuscular junction, In-vivo imaging, 3D image analysis, Segmentation, Registration, Semi-automatic workflow

## Abstract

**Background:**

Synapses, as specialised cell–cell contacts, allow for a faithful and controlled signal transmission between a neuron and a target cell. Presynapses, the sites of neurotransmitter release, form de novo throughout the development of an organism. Although this process is fundamental to the development and function of synaptic circuits, how developing neurons control number and distribution of individual synapses remains poorly understood. In-vivo imaging analysis of synapse formation at the neuromuscular junction of anaesthetised *Drosophila* third instar larvae allows for spatial and temporal resolution of the underlying molecular processes. However, high-throughput, comprehensive analysis are hampered by the manual and time-consuming imaging analysis methods applied hitherto. Here, we focus on the early presynaptic formation steps, that is, the presynaptic seeding, initiated by the formation of transient Liprin-*α*/SYD1 seeding sites, either stabilised or disintegrated over a time span of 30–90 min.

**Results:**

To investigate the dynamics of the Liprin-*α*/SYD1 seeding sites, we developed an automated analysis pipeline for 3D confocal images from in-vivo imaging at distinct time points to analyse fluorescently labelled presynaptic protein dynamics during early synapse formation. The workflow is realised in the data analysis software *Amira*, utilising the hierarchical watershed algorithm, and was designed for automatic processing with an option for manual proofreading. Compared to the previous 2D manual quantification, this automated approach provides a higher sensitivity in single Liprin-*α* seeding site detection in low-intensity areas and in regions of dense seeding sites. In addition, it substantially reduces the work time. To account for possible errors occurring in the automated processing, we implemented an additional proofreading step allowing for a manual correction of Liprin-*α* seeding site segmentation and assignment, thus greatly improving the analysis while only marginally increasing work time by 10% to a total work time reduction of 70% compared to the 2D manual analysis paradigm.

**Conclusion:**

The process of synaptogenesis underlies the general principles of locomotion, learning and memory formation. The developed fast and accurate semi-automated 3D workflow will provide a substantial progress in the analysis of this molecular process.

## Background

**Fig. 1:**
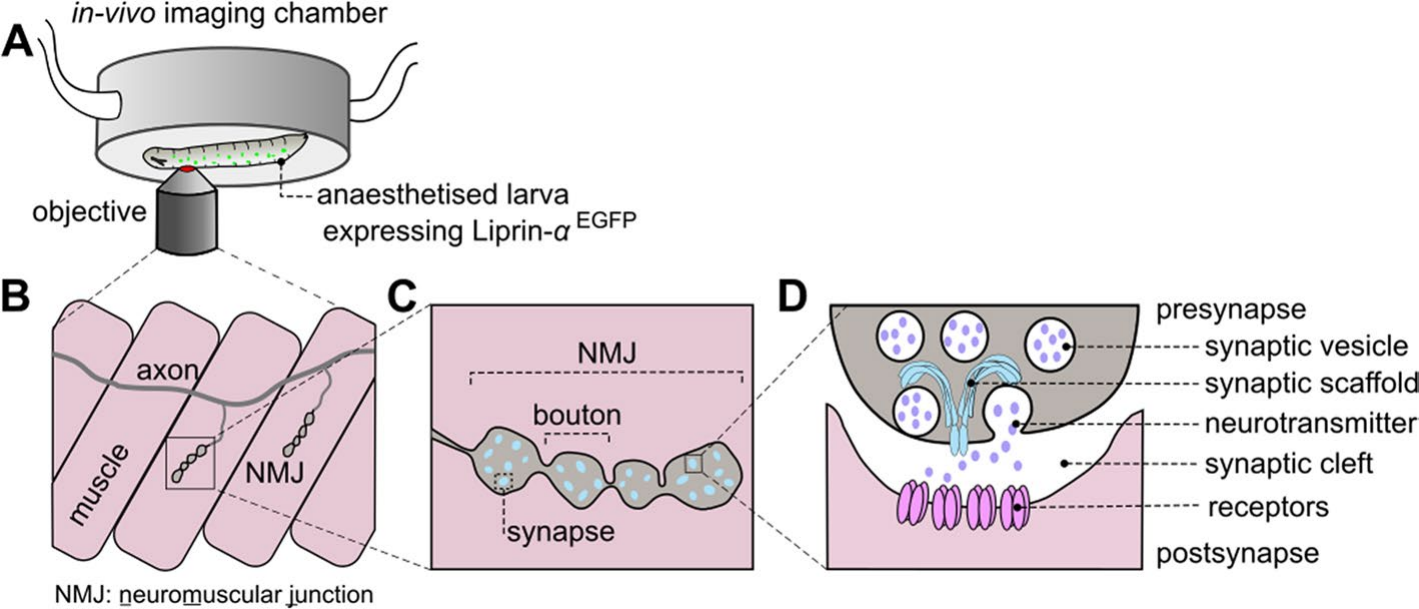
Introduction of the neuromuscular system. **A** Schematic presentation of in-vivo imaging of an anaesthetised larva expressing Liprin-*α*^EGFP^ in an in-vivo imaging chamber, **B** NMJs innervating the larval body wall muscle, **C** a single NMJ constituted of boutons containing single synapses, and **D** representation of a single mature synapse (cross section)

Synapses are highly specialised cell–cell contacts allowing for signal transmission between a neuron and a target cell. Both synapse formation and maturation are prerequisites for the development of neuronal circuits underlying cognition and behaviour. Although the molecular and cell-biological details of synapse assembly are extensively studied across species [14, 17, 23, 26, 31, 34, 42], the actual timeline of molecular pre- or postsynaptic assembly is less well characterised, as this requires technically challenging temporally resolved in-vivo imaging, preferably in a living organism. The developing neuromuscular junction (NMJ) of *Drosophila* third instar larvae represents a particularly suitable model to investigate the dynamics of molecular synaptogenesis over several hours in the anaesthetised transparent larva [2, 3, 12]. This system allows for the distinction of single synapses forming at the synaptic terminal connecting the presynaptic, neurotransmitter-releasing moto-neuron with the postsynaptic muscle, specialised to receive the neurotransmitter signal through postsynaptic glutamate receptors [11, 12, 19, 35] (Fig. 1). The NMJ-system profits from a well-defined and -described pre- and postsynaptic molecular architecture in addition to the accessibility of numerous established and characterised, genetically tagged, fluorescence-labelled protein-constructs designed for intravital imaging studies (Fig. 1).

**Fig. 2:**
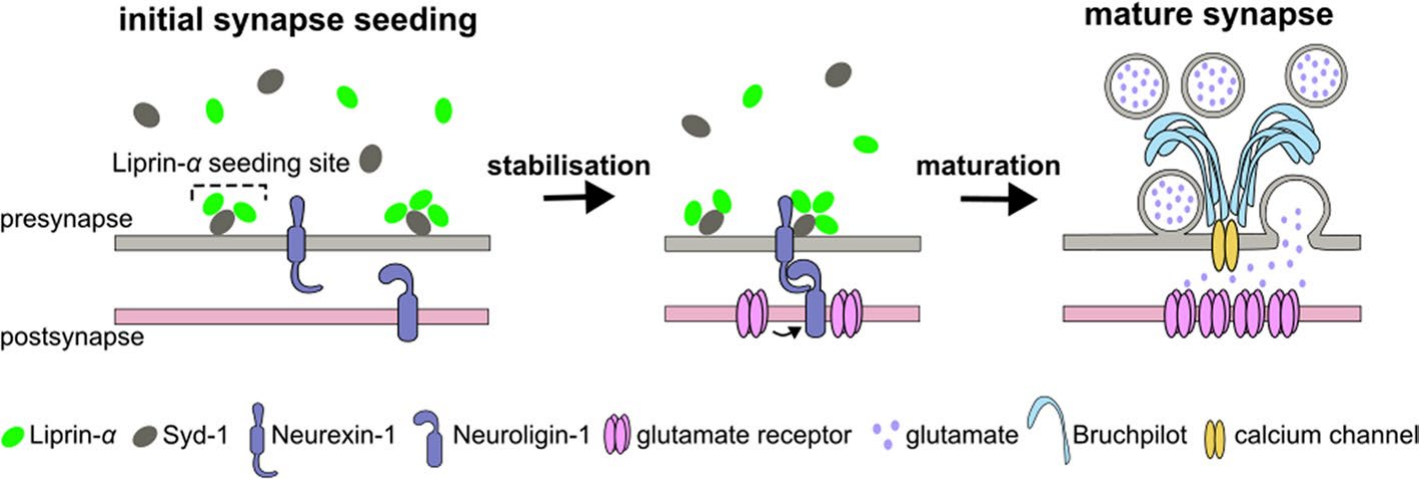
Model of synapse formation. For explanation see text

Previous laborious studies deciphered a fine-tuned molecular synaptic formation and maturation sequence at the NMJ (Fig. 2). In brief, at the individual synapse level, a stable early presynaptic protein complex of “seeding factors” forms to initiate synaptogenesis. This complex is composed of the scaffold protein Liprin-*α* (in vertebrates, SYD-2, Synapse-defective 2), stalled in place by the RhoGAP SYD-1 (Synapse-defective 1). SYD-1 is localised by the cell adhesion molecule Neurexin-1 (NRX-1), which is itself anchored through its postsynaptic binding partner Neuroligin-1 (NLG-1), both spanning the synaptic cleft [27, 28, 31, 32]. Once the early seeding complex is stably formed, postsynaptic glutamate receptors are recruited to the site of synapse nascency [7, 15, 27, 28, 33, 37] and finally signal back to the presynaptic scaffold to integrate later presynaptic scaffolding proteins like the ELKS/CAST homologue BRP (Bruchpilot), voltage-gated calcium (Ca2+) channels (VGCCs) and presynaptic release factors including UNC13 (Uncoordinated-13) to the now mature synapse [10, 15, 27].

**Fig. 3:**
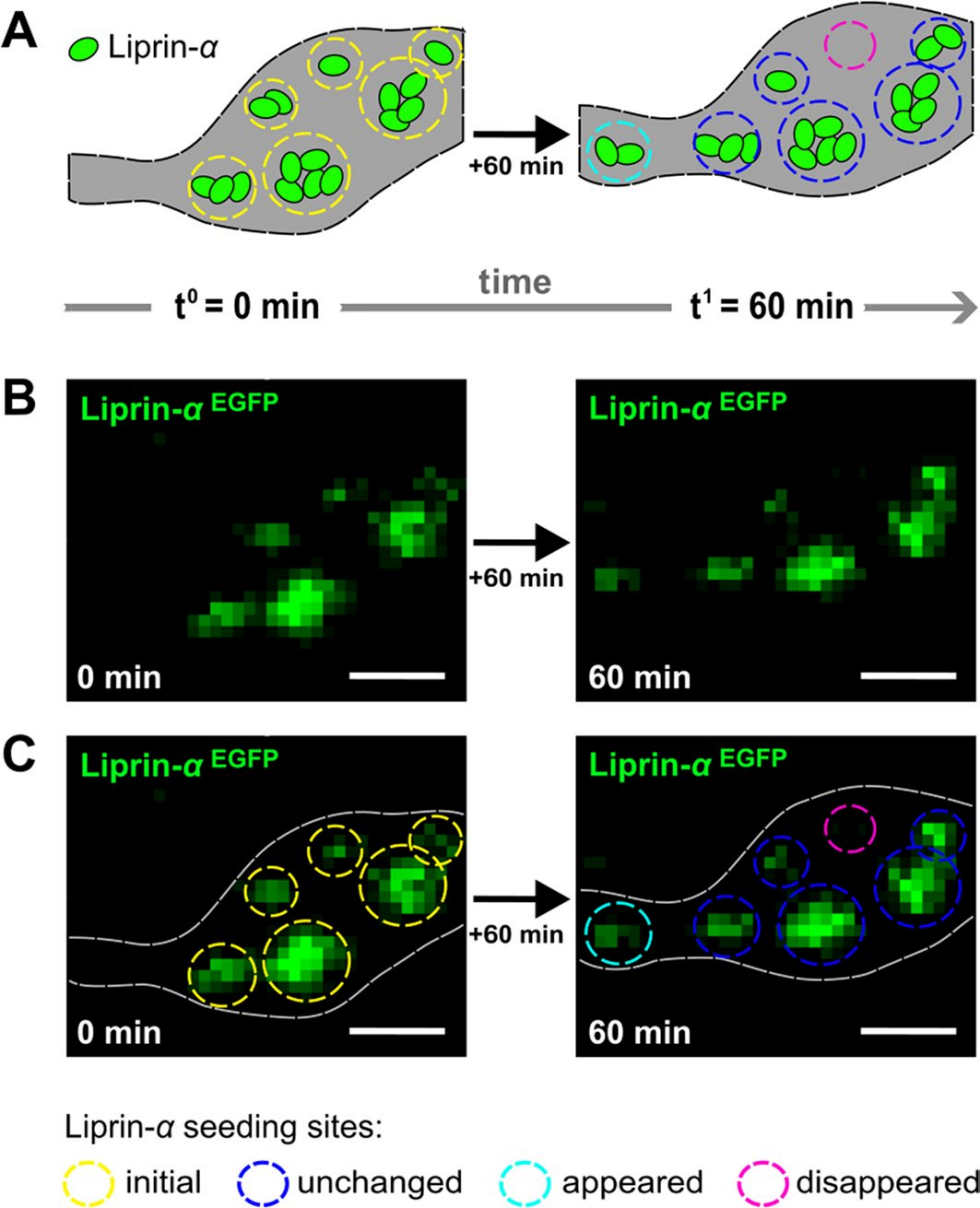
Quantification of Liprin-*α* seeding dynamics over time. **A** Schematic depiction of one bouton at time point t^0^ = 0 min shows several independent Liprin-*α* signals in distinct areas—the *initial* seeding sites. At t^1^ = 60 min, *unchanged*, *appeared* and *disappeared* Liprin-*α* seeding sites in comparison to the initially identified seeding sites at t^0^ = 0 min are determined. **B** Confocal in-vivo images at two respective time points of a representative NMJ bouton expressing Liprin-*α*^EGFP^ (scale bar 1 μm). **C** Same images as in B with outlined Liprin-*α* seeding sites (yellow circles: *initial*; blue circles: *unchanged*, turquoise circle: *appeared*, and magenta circle: *disappeared* seeding sites)

Interestingly, although Liprin-*α* initiates synaptogenesis, only a subfraction of the Liprin-*α*-positive seeding sites turn—in a non-predictable manner—into mature synapses, while others disappear within a time frame of 30–90 min [28] (Fig. 3). Both appearance and disappearance of Liprin-*α* seeding sites determine final number and spatial distribution of single synapses at the synaptic terminal, thus assigning synaptic strength and functional read-out of the neuromuscular junction. Liprin-*α* function in synapse seeding is not restricted to the neuromuscular system, but represents a common principle shown to function in both invertebrates [13, 29, 43] and vertebrates [14, 41, 42, 48] and has been recently shown to promote liquid–liquid phase separation (LLPS) during synaptogenesis [21, 24]. Defective synapse formation is associated with various neurodegenerative diseases [8, 46], and the detailed understanding of the temporal sequence and dynamics of early synapse formation could aid to further understand disease emergency.

Unfortunately, although of major importance for the understanding of molecular synaptogenesis, previous findings are based on a small number of synaptic terminals analysed due to the laborious, manual and, therefore, highly time-consuming image analysis [4, 15, 27, 28, 30, 32, 33, 37], based on 2D maximum projections, also losing all 3D information of the 3D boutons. To address these issues, here, for the first time, we introduce a pipeline that works directly with 3D confocal images from intravital imaging of distinct developmental time points. Using 3D images offers multiple advantages over the 2D analysis. In addition, a substantial workload reduction is achieved due to the semi-automatic nature of the method. The prototypical workflow was developed within the 3D visualisation and data analysis software Amira [40] using *C++*, but it could also be implemented within other environments, e.g. *SimpleITK* [22], using *Python*.

## Materials and methods

### Fly husbandry and genetics

*Drosophila melanogaster* strains were reared under standard laboratory conditions at 25 °C and 70% humidity on semi-defined medium (Bloomington recipe) [41]. Flies expressed the previously described protein-construct UAS Liprin-*α*^EGFP^  [26] using the motoneuronal *ok6*-Gal4 driver (PGawBOK6; [1]). In-vivo imaging was conducted with heterozygous animals at 25 °C. For all experiments, both male and female larvae were used for analysis.

### In-vivo imaging

Imaging of intact, living *Drosophila* larvae expressing Liprin-*α*^EGFP^ through the UAS-Gal4 system was performed as previously described [4, 33]. In brief, young third instar larvae were anaesthetised with Desflurane (Baxter) for 2 min in a custom build chamber until all muscle contraction stopped. Confocal image stacks were acquired from muscles 26 or 27 in segments A1 to A3 of the larvae at room temperature with a HC PL APO CS2 63x/1.40-N.A. oil objective (Leica Microsystems) in a Leica DMI 6000/SP8 (Leica Microsystems) microscope. EGFP was excited using a solid-state laser at 488 nm. Fluorescence detection was set with a Hybrid detector between 500 and 530 nm for EGFP. Digital gain was set to a maximum of 45%. Larvae were subsequently transferred to a food plate to reanimate and after 30 or 60 min re-anaesthetised in the in-vivo chamber for the 30 or 60 min time point image acquisition. Larvae were only used for subsequent analysis, if they revived after the last anaesthetisation step. The resulting images have limited resolution in Z axis which led to an anisotropic voxel size of $0.1 \times 0.1 \times 0.3$ μm^3^.

### Processing workflows

**Fig. 4:**
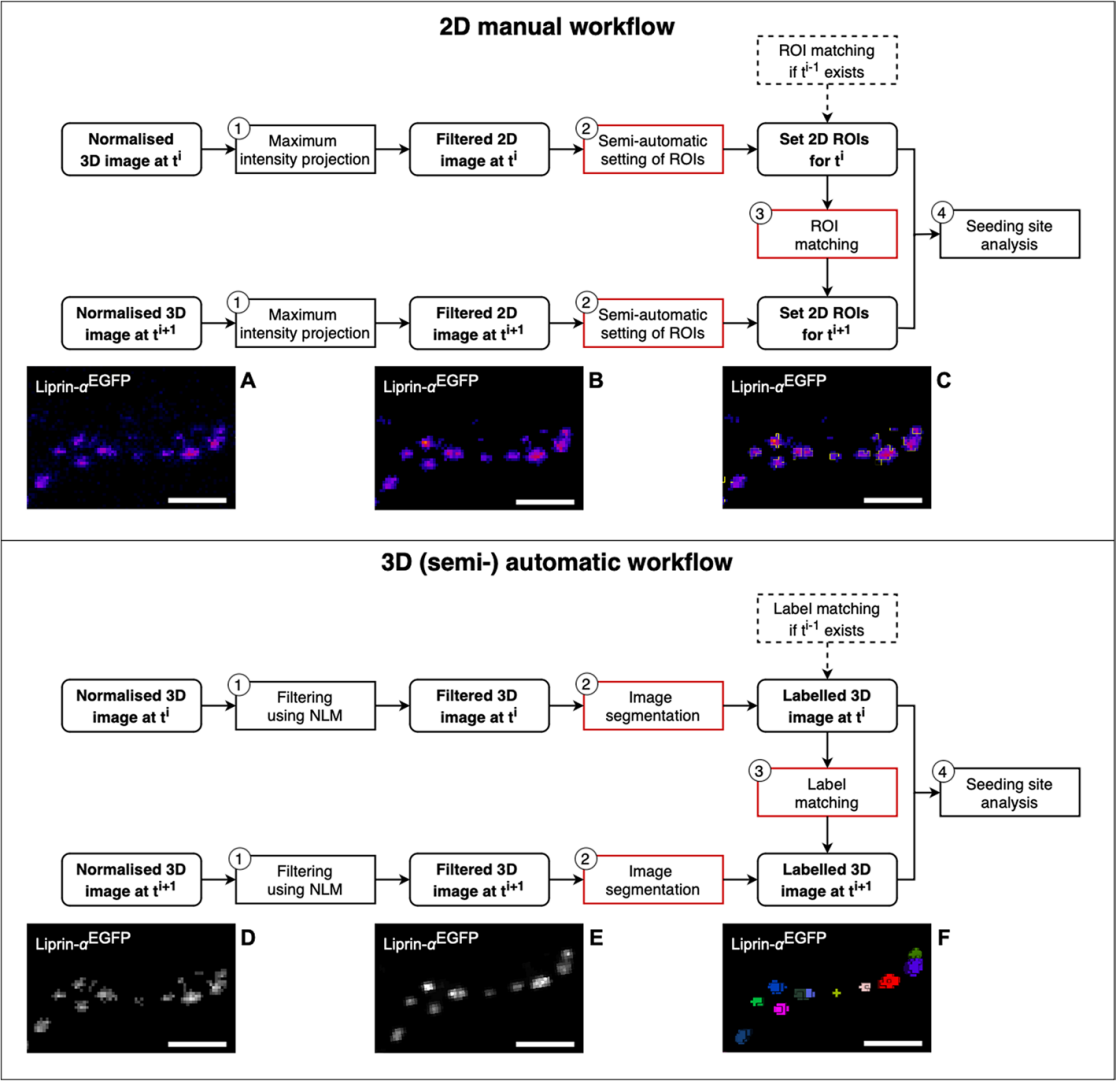
2D and 3D workflows. Images within the workflows visualise intermediate results (scale bar 2 μm). **A** Raw image stack displayed in *FIJI* in a colour scale indicating the pixel intensity value. **B** 2D image after background removal and maximum intensity projection. **C** 2D image with ROIs marking individual seeding sites. **D** Normalised image stack (cross-section) displayed in Amira. **E** 3D image (cross-section) after denoising. **F** Segmented 3D image with labels representing single seeding sites. Steps with red frames may require manual corrections in case of the 3D semi-automatic workflow

An approach to enable a better understanding of the maturation of presynapses is to study the dynamics of Liprin-*α* seeding sites. This is based on the identification and separation of individual seeding sites as well as their tracking over individual time points from the acquired confocal images. A standard protocol for this analysis is adapted from [5]; it requires dimension reduction (from 3D to 2D images) and a substantial amount of labour intensive work. To circumvent shortcomings of the 2D workflow and reduce manual labour, we introduce a semi-automatic workflow working directly with 3D images (Fig. 4). Implementation of both workflows is described in the appendix.

All workflows were executed on a personal computer running a 64-bit Windows 11 operating system, equipped with an Intel Core i5-11500 CPU with 64 GB of RAM and an NVIDIA GeForce RTX 3060 graphics card with 8 GB VRAM.

#### Normalisation

Before applying either the 2D or 3D workflow, normalisation is performed to preprocess the original image stack. To compute the mean intensity background value $\overline{I_b}$, three circular, representative ROIs are manually chosen, avoiding tracheae and axon region (see Fig. 10). The gray value of each voxel is then adjusted as described in the following equation:

1 $$\begin{aligned} {I_{adj} = \frac{255}{255 - \overline{I_b}} \cdot (I-\overline{I_b})} \end{aligned}$$

where:

- $I_{adj}$ is the adjusted intensity value of the voxel,
- *I* is the original intensity value of the voxel, and
- $\overline{I_b}$ is the mean intensity value of the selected background region.

Normalisation is applied separately for each image. This might theoretically cause major differences in amplification in the images within a single dataset. However, as the $\overline{I_b}$ value is typically low and does not differ significantly between the images within a single dataset, the amplification difference should be negligible.

#### 2D workflow

In order to allow for a direct comparison between the established 2D workflow and the proposed 3D workflow, here, we briefly recall the previously used method based on 2D projections of the 3D image data. The 2D workflow (Fig. 4, top) consists of the following steps:

1. Maximum intensity projection
2. Segmentation and ROI selection
3. ROI matching
4. Seeding site analysis

These steps integrate both semi-automatic procedures and manual refinements (highlighted in red in Fig. 4, top) to achieve optimal results.

##### Maximum intensity projection

After normalisation, the image is projected into 2D, using maximum intensity projection, yielding a single 2D image that is used for further analysis.

##### Segmentation and ROI selection

Segmentation of the 2D projection image begins by applying a threshold to narrow down on Liprin-*α* signals, where the lower threshold is set to preserve essential features and exclude irrelevant intensities. The image is thereby transformed into a binary mask, which is further used to create an overlay with the original intensity image, resulting in a new image in which all the pixels below the specified threshold are set to 0. This thresholded intensity image is then segmented automatically by identifying regions around local maxima that are overlaid with the maximal intensity projection image. Thresholding values as well as maxima detection parameters need to be determined for each dataset individually. The segmented particles are identified as potential regions of interest (ROIs), i.e. Liprin-*α* seeding sites.

Manual review ensures the accuracy of selected ROIs by merging, splitting, or adjusting regions as necessary.

##### ROI matching

*Unchanged*, *appearing*, and *disappearing* Liprin-*α* seeding sites must be unambiguously distinguished. To ensure this, ROI sets are manually tracked across different time points. The same ROIs over time are manually assigned the same identifier. This labelling facilitates consistent tracking and categorisation throughout the analysis.

##### Seeding site analysis

A comparative analysis between time points is performed. This way, we identify and analyse the number of Liprin-*α* seeding sites that are *unchanged*, are *appearing* or have *disappeared*. Additionally, quantitative data, e.g. area, integrated density, and mean pixel intensity of each labelled region, can be extracted.

#### 3D workflow

To take full advantage of the 3D information obtained from in-vivo imaging and to reduce the manual labour, we developed a 3D workflow. This workflow can be used fully automatically but it also allows for semi-automatic proofreading to further improve the results. The 3D workflow (Fig. 4) consists of the following steps:

1. Image filtering
2. Image segmentation
3. Label matching
4. Seeding site analysis

All the steps can be performed automatically, but may require the user to adjust the parameters or to perform manual correction for best results. A graphical flowchart illustrating each step of the 3D workflow is provided in Fig. 13 in the appendix.

##### Filtering

Filtering removes undesired noise from the raw images. In the current implementation of the workflow, the non-local means filter (NLM) [16] is utilised. NLM performs favourably compared to more traditional filtering methods, as it preserves edges and fine details by averaging voxels with similar neighbourhoods instead of computing an average of nearby voxels.

##### Segmentation

Segmentation is performed using the hierarchical watershed algorithm [9]. The drawback of a traditional watershed algorithm is that the noise in the image can cause an over-segmentation of the image. Using the hierarchical watershed algorithm circumvents this issue by allowing the user to merge segmented regions based on the persistence of the segmented regions. To focus on voxels with high intensity, an intensity range is used to facilitate the segmentation, such that all voxels outside that range will be labelled as background. Besides improving the segmentation results, restricting the range of intensity values contributes greatly to the computational efficiency [6]. Due to photobleaching during the imaging process, the range has to be defined separately for each image. This can be done either manually or using an automated thresholding method (e.g., Otsu’s method [25]).

Manual correction is possible if the user finds the segmentation algorithm’s output unsatisfactory. Over-segmented labels have to be merged and under-segmented labels need to be split (Fig. 12). Labels representing noise need to be removed.

The final result of the segmentation is an image where each seeding site is represented with a label carrying a unique identifier (in contrast to the 2D manual workflow where ROIs represent seeding sites with unique identifiers), corresponding to the physical constrains of the seeding site.

##### Label matching

Muscles grow and can move between two consecutive imaging steps, hence, matching regions in the corresponding images may not overlap. To compensate for this, 3D image registration is applied prior to matching the labels. Linear registration, which allows for translation, rotation, scaling and shearing, is applied. As similarity metric, normalised mutual information (NMI) is used. Since the images of consecutive time points are of similar size and are oriented similarly, no pre-alignment is required. The image at time point t^i^ is aligned to the image of t^i+1^. Registration algorithms typically compute the optimal transformation of the moving image without actually transforming the image, that is, without sampling it onto a new grid. Consequently, the moving image still resides in its original coordinate system. To facilitate an easy overlap computation where one simply counts the number of voxels of a certain label of the moving image that overlap with a label of the fixed image, the labels of the registered image are sampled onto a new grid with the same coordinates and dimensions as the grid of the fixed image, that is, the reference.

As the images of time points t^i^ and t^i+1^ are segmented independently from each other, the same object in different time points usually gets assigned different label IDs. To address this problem, a label-matching algorithm was developed. It takes the labels of two segmented images (from time points t^i^ and t^i+1^) as input and reassigns new label IDs to the image of time point t^i+1^. This is done by first computing the overlap between all labels in both images. Then, the segment in t^i+1^ with the largest overlap with a segment from t^i^ is assigned the same label as that segment (in t^i^) and is considered as *unchanged*. The label that was assigned is marked as handled and the next largest label overlap is computed. This is repeated until no more labels can be assigned. If a segment from t^i^ has no overlapping segment in t^i+1^, or if its overlap is not the largest, it is considered to have *disappeared*. Similarly, if a segment in t^i+1^ has no overlap with any segment from t^i^, it is classified as *appeared*. In case the user notices mismatched labels, manual corrections are possible.

It should be noted that image registration is performed by aligning $t^i$ to $t^{i+1}$, while label matching is applied in the reverse direction, from $t^{i+1}$ to $t^i$. The following sequence is followed during the analysis: First, individual seeding sites are analysed at $t^i$ (“Seeding site analysis”(i) section), followed by registration of $t^i$ to $t^{i+1}$ and label matching of $t^{i+1}$ to $t^i$. Next, seeding sites at $t^{i+1}$ are analysed (“Seeding site analysis”(i) section), and finally, seeding sites are compared across the two time points (“Seeding site analysis”(ii) section). This ensures two things: (1) the first image keeps its original labels while all other images will have their labels reassigned; (2) analysis of seeding sites in individual images will always be extracted from a non-deformed image, ensuring accuracy of the information.

##### Seeding site analysis

Two types of seeding side analysis can be performed:

(i) Analysis of seeding sides in individual time points: The following information about the segmented (labelled) Liprin-*α* seeding sites can be extracted from each image (time point) separately: diameter (sites are assumed to be spherical), anisotropy, elongation, flatness, accumulated intensity values, mean intensity values. It must be noted that the mentioned information is extracted from the non-registered images as the registration may contribute to minor errors due to image deformation.
(ii) Comparison of seeding sites between time points: In addition, we can extract further information by comparing the labelled images of time points t^i^ and t^i+1^ w.r.t. the matched segmented Liprin-*α* seeding sites. The result is a spreadsheet with information, which segments have been matched, which have disappeared and which have appeared. To assess quantitative information from these results, the following additional information is provided:
  For *unchanged* segments: the label ID as well as the absolute and relative volume overlap of the seeding sites from one time point to the next.
  For* disappeared* segments: the label ID of the seeding site present in t^i^ but no longer present in t^i+1^.
  For *appeared* segments: the label ID of the seeding site present at t^i+1^ but not present in t^i^.

**Visualisation**

**Fig. 5:**
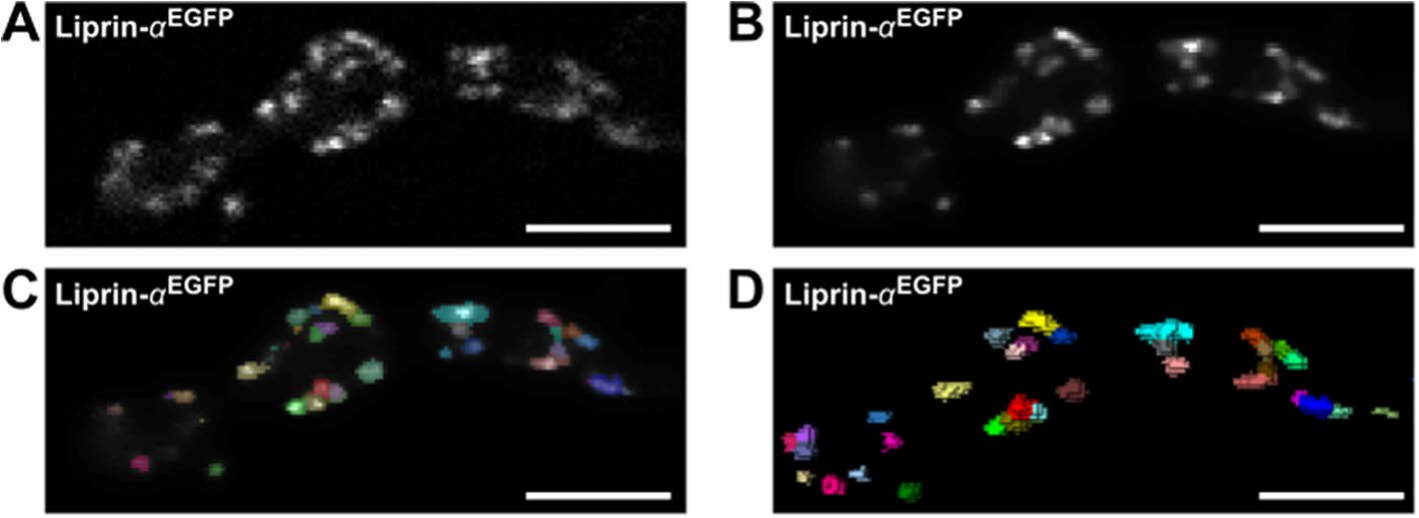
Visualisation of intermediate results using the Amira software. A single NMJ expressing Liprin-*α*^EGFP^ shown using different visualisation methods (scale bar 4 μm). A single slice of the raw image stack is displayed in 2D before (**A**) and after filtering (**B**). The same filtered image can also be displayed in 2D (single slice shown) overlaid with the segmentation and blended with the intensity values (**C**). The image segmentation is visualised in 3D using voxelised volume rendering (**D**)

Visualisation is an integrative part of the workflow. When necessary, users can visualise any intermediate results, especially when tuning module parameters or performing proofreading. For displaying raw or filtered images, showing 2D data slices is recommended. This is particularly useful for evaluating filtering or registration outcomes. Segmentation results can be proofread effectively by overlaying the segmented data with the raw or filtered images using blending techniques. For labelled data, volume rendering is the preferred visualisation method. Figure 5 shows different ways of visualising the data.

## Results

This section provides the results of a quantitative analysis of the 3D automatic and semi-automatic workflows.

### Registration

**Fig. 6:**
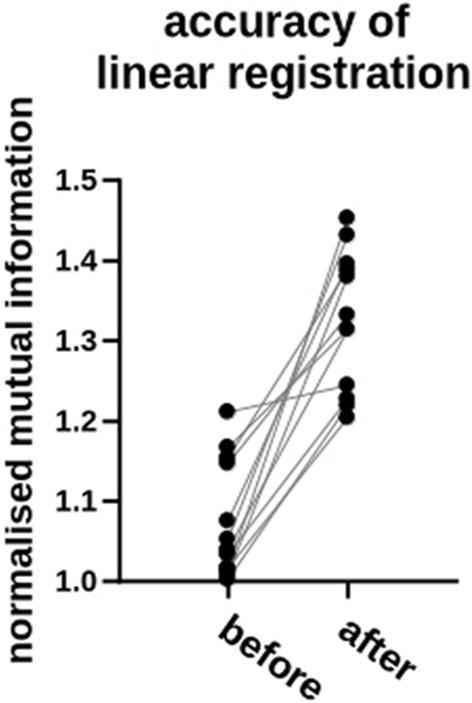
Comparison of the NMI metric for 12 samples before and after the registration

The success of the registration has been evaluated by comparing the optimisation metric (normalised mutual information, NMI) before and after the registration. The results for 12 examples can be seen in Fig. 6.

For all datasets, as expected, the score after registration is higher than before the registration process.

### Manual correction rates in 3D semi-automatic workflow

In case of utilising the 3D semi-automatic workflow, the user needs to manually correct the outcome of the segmentation algorithm. To gain an understanding of segmentation accuracy we have tracked the operations envoked during the correction step.

Operations can be of the following types:

(i) *Delete label*: In case of noise being detected, the label has to be deleted.
(ii) *Split label*: In case of under-segmentation, the label has to be split into two.
(iii) *Merge labels*: In case of over-segmentation, multiple labels should be merged.
(iv) *Assign ID*: In case the same seeding site in two distinctive time points has been assigned different label IDs, they have to be matched manually.

**Fig. 7:**
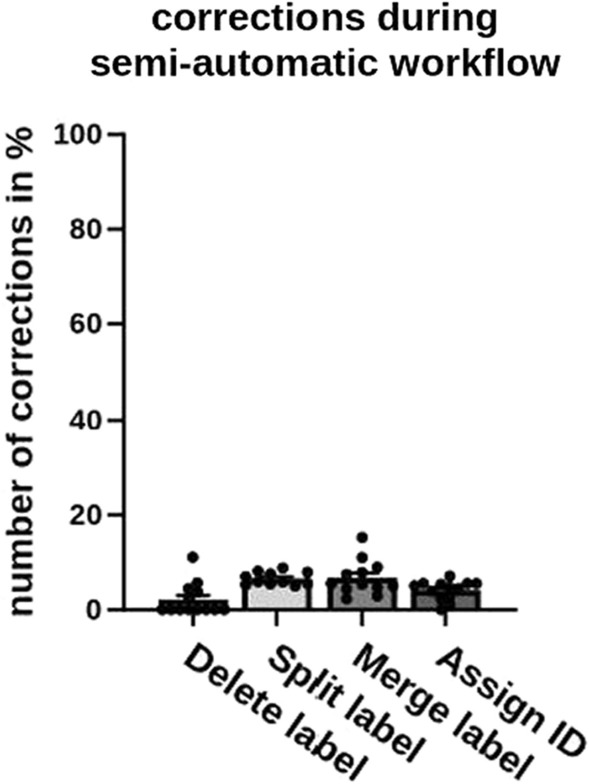
Rates of correction during semi-automatic workflow. Number of corrections normalised to total initial count of labels in the segmentation results. Exact counts: *delete label: 2.14%*, *split label: 6.61%*, *merge labels: 6.74%*, *assign ID: 4.12%*

Operations i–iii are performed on each image after segmentation, while operation iv is performed after the label matching has been applied. A single operation tracking experiment consists of corrections after segmenting two images (same dataset, two time points) and matching their labels. It was performed for 13 datasets, the results of which can be found in Fig. 7. On average labels have to be split, merged or assigned for approximately 7% of the labels, while they have to be deleted less often (2%). Label IDs have to be manually assigned for 4% of the labels.

### Workflow outcome results

Clusters of Liprin-*α* proteins, here called seeding sites, form during early synapse formation at the presynaptic membrane and either become stabilised by other protein–protein interaction to form mature synapses or dissolve if the protein–protein interactions cannot be formed. Here, we developed a 3D quantitative analysis workflow of these in-vivo molecular changes over time. The workflow allows us to first quantify the *initial number* of seeding sites, that is, all seeding sites at some initial time point t^0^. Then, at subsequent time points t^1^, …, t^n^, we can distinguish three seeding site groups, always with respect to the previous time point: (i) newly formed seeding sites (*appeared*), (ii) dissolved seeding sites (*disappeared*), and (iii) *unchanged* seeding sites, which are present in both the current and the previous time point (Fig. 3).

**Fig. 8:**
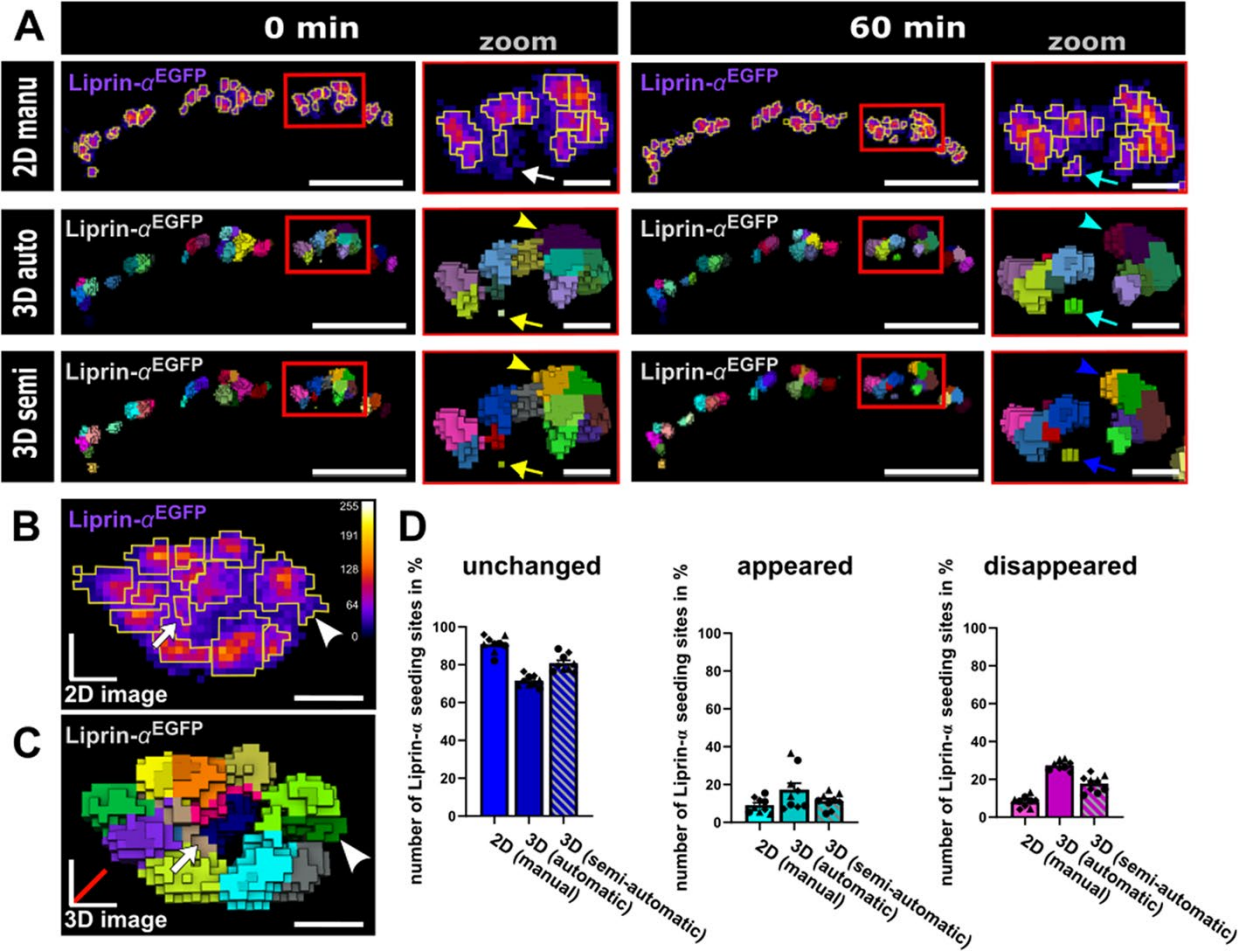
Comparison of 2D manual workflow and 3D automatic/semi-automatic workflows. **A** In-vivo confocal images of a representative neuromuscular terminal expressing Liprin-*α*^EGFP^ at 0 min (left column) and 60 min (right column) (overview scale bar 4 μm, zoom according to red rectangle scale bar 1 μm). Upper row, 2D image with manual segmentation, middle row 3D segmentation using automatic workflow, last row 3D semi-automatic workflow. Arrows and arrowheads mark examples of different Liprin-*α* seeding sites identification (see text for more information). The colours of the segments are randomly assigned to the different seeding sites. **B** 2D segmentation of the Liprin-*α*^EGFP^ signal of one bouton at 0 min processed with the 2D manual workflow. The colour scale indicates the pixel intensity value. **C** The same image data as in B processed with semi-automatic segmentation (3D). Arrows and arrowheads indicate differences in seeding site identification (see text for detailed explanation, scale bar 1 μm). **D** Comparison of the number of detected Liprin-*α* seeding sites that are unchanged, disappeared or appeared at 0 min and 60 min of manual 2D workflow, automatic and semi-automatic 3D workflows. The manual workflow was performed by 3 different experimenters (circle = experimenter A, triangle = experimenter B, rectangle = experimenter C); unchanged: 2D (manual) = 90.76% ± 1.42%, 3D (automatic) = 71.43% ± 0.96%, 3D (semi-automatic) = 80.75% ± 1.50%; appeared: 2D (manual) = 9.07% ± 1.31%, 3D (automatic) = 17.09% ± 3.64%, 3D (semi-automatic) = 11.28% ± 1.36%; disappeared: 2D (manual) = 8.22% ± 0.97%, 3D (automatic) = 27.40% ± 0.93%, 3D (semi-automatic) = 17.74% ± 1.47%; N = 9 NMJs

**Fig. 9:**
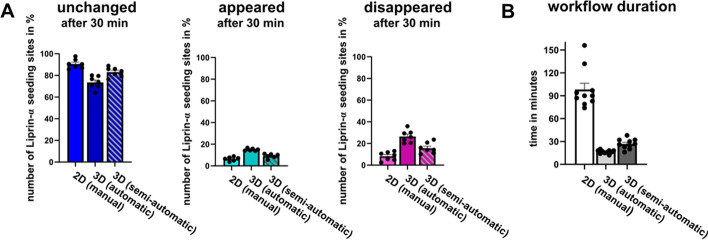
Comparison of 2D manual workflow and 3D automatic/semi-automatic workflows. **A** Comparison of the number of Liprin-*α* seeding sites that are unchanged, disappeared or appeared at 0 min and 30 min detected with manual 2D workflow, automatic and semi-automatic 3D workflows; unchanged: 2D (manual) = 90.52% ± 1.61%, 3D (automatic) = 73.57% ± 2.18%, 3D (semi-automatic) = 83.07% ± 1.65%; appeared: 2D (manual) = 6.43% ± 0.80%, 3D (automatic) = 14.97% ± 0.47%, 3D (semi-automatic) = 9.12% ± 0.84%; disappeared: 2D (manual) = 8.24% ± 1.52%, 3D (automatic) = 26.43% ± 2.18%, 3D (semi-automatic) = 15.66%  ± 1.88%; N = 7 NMJs. B) Time requirements for each workflow per NMJ; manual = 98.20 min ± 25.71 min; automatic = 16.40 min ± 0.73 min; semi-automatic = 26.90 min ± 2.03 min; N = 10 NMJs

**Fig. 10:**
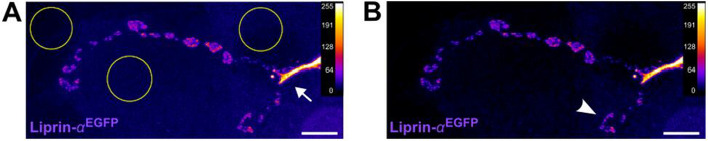
Background correction during the 2D manual workflow. **A** An in-vivo confocal image in maximal intensity projection shows two NMJs expressing Liprin-*α*^EGFP^ grown from one axon (white arrow). The mean background intensity is measured in three similarly sized areas that should not overlap with NMJ nor axon. **B** The result shows less background noise. Note that axon and additional NMJ (white arrowhead) will be removed in following steps using the freehand tool. The colour scale indicates the pixel intensity value. Scale bar 5 μm

**Fig. 12:**
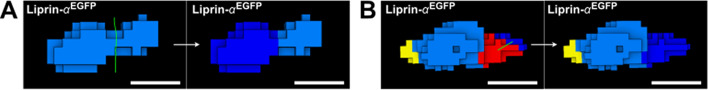
Splitting and merging labels. Representative areas of Liprin-*α*^EGFP^-positive signal in an NMJ shown in 3D segmentation (scale bar 0.5 μm). **A** A label is split by drawing a line at the desired split location. **B** Merging two labels requires a scribble over the label border

**Fig. 13:**
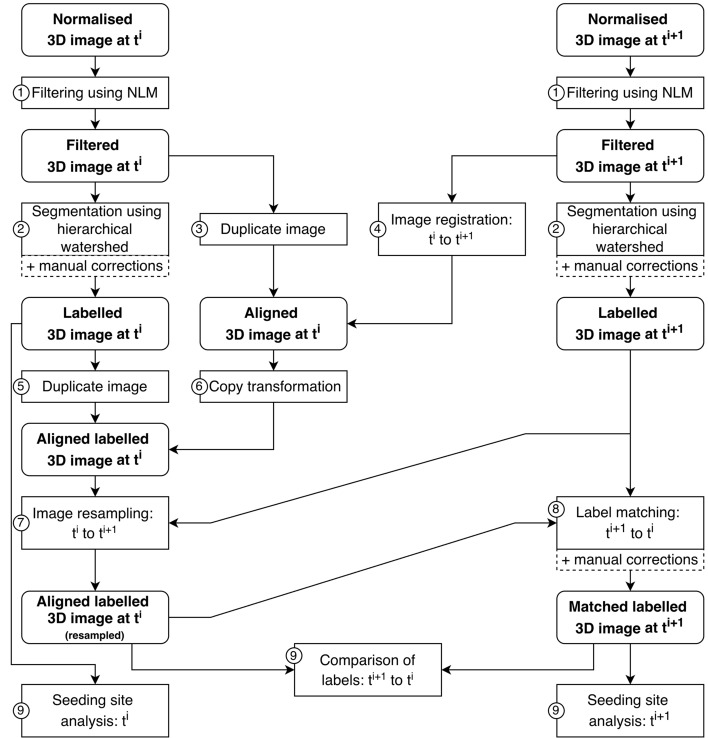
Detailed graphical flowchart of the 3D workflow. Each operation of the workflow (represented as rectangles) corresponds to a module used in Amira. Numbers next to the operations suggest the order of operations. Rectangles with dashed lines represent operations to be envoked only with semi-automatic workflow

To test the new 3D workflow, we analysed Liprin-*α* seeding sites in 16 datasets of the neuromuscular junction of living third instar *Drosophila* larvae. Nine datasets contain time points t^0^ = 0 min and t^1^ = 60 min, while seven datasets are evaluated at time points t^0^ = 0 min and t^1^ = 30 min, see Figs. 8 and 9. All datasets were evaluated using the 2D manual approach, the 3D automatic approach, and the 3D semi-automatic approach (Fig. 4). Manual 2D analysis for the datasets comprising of time points t^0^ = 0 min and t^1^ = 60 min was performed by three different experimenters. Manual 2D analysis for the datasets comprising of time points t^0^ = 0 min and t^1^ = 30 min, the 3D automatic and semi-automatic analyses were performed by the same experimenter.

Firstly, we compared the original 2D manual workflow based on maximum projection to the 3D automatic workflow (Figs. 4 and 8A–F). Both methods capture most of the detected Liprin-*α* seeding sites correctly (Fig. 8A), however, the 3D automatic analysis shows a higher degree of sensitivity in the detection of single Liprin-*α* seeding sites with both low intensities and regions dense with seeding sites (Fig. 8A–C). Figure 8A shows a representative case for a low intensity Liprin-*α* seeding site that was not detected with the 2D manual workflow (the white arrow indicates the undetected seeding site at t^0^ and the blue arrow indicates the corresponding site at t^1^), while the 3D workflows detect the Liprin-*α* seeding site correctly at t^0^ (yellow arrows at t^0^). Note that the correct detection of all seeding sites at t^0^ (*initial* seeding sites) is essential for the correct classification of seeding sites at t^1^, as otherwise not identified seeding sites at t^0^ will be misclassified as *appeared* instead of *unchanged*, with substantial consequences for the biological interpretation of the data. The 3D automatic workflow considerably decreased the mean working time from 98 min to 16 min per NMJ (Fig. 9B). We observed that compared to the 2D manual workflow, the 3D automatic quantification resulted in 19% reduced *unchanged* seeding sites (Fig. 8D left panel) and higher representation of *appeared* (Fig. 8D middle panel) (8%) and *disappeared* (Fig. 8D right panel) (19%) seeding sites with time points set at t^0^ = 0 min and t^1^ = 60 min. Results displayed in Fig. 9A compare the classification of the seeding sites in a similar manner, but for the time points t^0^ = 0 min and t^1^ = 30 min. The *unchanged* seeding sites appear to be classified less often with the 3D automatic workflow (17% reduction). Appeared seeding sites are classified 8% more often and the disappeared ones 18% more often.

When investigating the reasons for these differences, we identified two types of errors. The first one is a mismatching of the seeding sites in consecutive time points. This is the result of the segmented seeding sites not overlapping even after image registration. A representative mismatched case is depicted in Fig. 8A (middle panel), where at t^0^ = 0 min the Liprin-*α* seeding site is detected (yellow arrow), but not registered and matched to the corresponding seeding site at t^1^ = 60 min (blue arrow), falsely classifying the seeding site at t^1^ as an *appeared* instead of an *unchanged* site. We observed that mismatching of seeding sites mostly occurred with very small seeding sites that, with our 3D automatic workflow, are more often detected than with the 2D manual one. Because of their small size, already a minor movement might result in the seeding sites not overlapping anymore in consecutive time points. Reasons for this are subtle movements of the larval muscle harbouring the NMJ, consequently changing seeding site position and orientation. Additionally, the NMJ itself undergoes developmental changes over time, e.g. increase in bouton size and localisation [27]. The second type of error occurred when proximate Liprin-*α* seeding sites were not correctly separated and instead segmented as one fused seeding site (Fig. 8A, middle panel, yellow arrowhead at t^0^), while at the other time point the seeding sites were correctly split (Fig. 8A, middle panel, blue arrowhead at t^1^). Again, this leads to a misclassification of seeding sites.

We therefore refined the 3D automatic workflow into a 3D semi-automatic workflow by implementing a proofreading step (Fig. 4, bottom, included red frames) to circumvent the above-described drawbacks of the 3D automatic workflow. This manual correction step allows for correction of the seeding site segmentation, resulting in accurate matching of Liprin-*α* seeding sites over time (Fig. 8A, lower panel). The yellow and blue arrows mark the correctly matched seeding sites. The yellow and blue arrowheads indicate the correct separation of seeding sites over time (Fig. 8A, lower panel), where the formerly fused seeding sites at t^0^ = 0 min were manually separated. Consequently, all seeding sites should now be classified into the correct groups (*appeared*, *disappeared* and *unchanged*), as quantified in Fig. 8D, comparing 2D manual and 3D semi-automatic workflows at t^1^ = 60 min. The same trend can be observed for the seeding sites analysed at t^0^ = 30 min, as seen in Fig. 9A. Although the additional data curation of the 3D semi-automatic workflow requires additional time (ca. 10 min), the overall time retrenchment of approximately 70 min (70%) compared to the 2D manual approach is immense (Fig. 9B).

Importantly, both 3D workflows support extracting additional biologically relevant data about the Liprin-*α* seeding sites, which can be used to investigate the underlying molecular mechanisms of seeding site formation and maturation (Table 1), e.g. diameter and mean pixel intensity of the seeding sites.

**Table 1:** Information extracted from three raw images at 0 min using the 3D workflows

NMJ	Number of seeding sites		Mean seeding site diameter (in μm)		Mean voxel intensity (per seeding site)	
	Auto	Semi	Auto	Semi	Auto	Semi
1	58	43	0.80	0.83	45.29	45.32
2	96	87	0.70	0.73	23.63	23.74
3	68	63	0.64	0.66	39.17	39.28

## Discussion

The developed 3D workflow aims to improve the established, work-intensive protocol for analysis of NMJs in *Drosophila* larvae [5]. It is based on registration, segmentation and segment-overlap measurement. It can work fully automatically, or semi-automatically by providing the tools for manual correction of intermediate results. According to the authors’ knowledge, this is the first publication dealing specifically with the analysis of *Drosophila* larvae NMJs in 3D.

Seeding sites in NMJs do not exhibit motility and it is the muscle where the seeding sites are located that grows and deforms over time. The focus of the neurobiological research requires an analysis of seeding site turnover (i.e., classification as *unchanged*, *appeared* and *disappeared* in distinctive time points), along with their morphological properties. However, the described problem shares similarities with object tracking problems and it could be approached this way. The following paragraphs discuss other available tools that could potentially be used for the purpose described in this paper.

*NMJ Analyser* is a NMJ specific tool implemented in ImageJ [39]. It attempts to quantify zebrafish NMJ morphology from confocal microscopy images. It allows for segmentation of NMJs into individual seeding sites, counts of pre- and post-synaptic puncta and automatically determines their co-localisation. However, the analysis it provides is based on Z-projections and individual slices and does not support 3D analysis, nor processing time series.

*TrackMate* is a popular tool for segmentation and tracking of 2D/3D blobby objects [44]. The objects are treated as points and are detected utilising spot detection algorithms (Difference-of-Gaussian or Laplacian-of-Gaussian). Its tracking algorithm for prevention of splitting and merging of events, could be potentially appropriate for our task. However, as the cost to link two spots is computed solely based on their respective distances, in practice this means that repositioning the sample or muscle growth might break the algorithm. As image registration is not provided by *Trackmate* it will be necessary to apply it a priori.

The commercial tool *Imaris* also provides a dedicated object tracking tool. Similarly to *TrackMate*, it focuses on the trajectory and not the stabilisation, formation and loss of small 3D structures. It offers multiple 3D tracking algorithms including Brownian motion, autoregressive motion, connected components and lineage.

*CellProfiler* is another multi-purpose tool with built-in options for image segmentation in object tracking [20]. Compared to the proposed workflow, it provides only limited 3D segmentation options and does not provide a user interface for segmentation correction. It provides distance-based nearest neighbour object tracking and overlap-based tracking (similar to our implementation). Distance-based tracking assumes slow object movement, low object density and no large drift or deformation, making it inapplicable for our task. Overlap-based tracking requires image registration to give robust results, which is not provided in the tool.

*3DeeCellTracker* provides an approach for object segmentation and tracking using deep learning [47]. After neural network-based segmentation, object tracking is implemented using a feed-forward network that predicts cell position based on spatial patterns of cells in previous and current images. We believe that this tool could provide a good alternative for our tool given that enough training data for segmentation will be provided.

Overall, our tool delivers full functionality needed for analysis of the posed neurobiological question. Firstly, it provides the crucial ability for 3D analysis, as the volumetric and morphological information about the seeding sites is needed. Furthermore, as muscle movement and growth are expected in our samples, image registration is a prerequisite to ensure that object correspondence across time reflects true biological turnover events rather than apparent positional changes caused by tissue deformation. Lastly, our workflow does not include machine learning based algorithms and therefore does not depend on training data. As a result, it works out of the box, without the need for training data. This is particularly advantageous given that the availability of training data is one of the major bottlenecks in many modern image analysis tools [38].

### Guidelines for workflow application

The 3D workflow begins with image filtering, which should be applied conservatively to preserve signal–background gradients. Subsequent segmentation must be performed consistently across all images in the dataset, requiring the user to carefully adjust and optimise parameters to ensure accurate results. In both cases, visualisation modules can be used to proofread the results of filtering and segmentation.

When the user decides to apply the semi-automatic approach, the segmentation must be manually corrected. This is the most time-consuming part of the workflow and takes almost half of the processing time. In Fig. 8A (middle panel, t^0^), the previously mentioned under-segmentation of the seeding sites can be seen (yellow arrowhead). The parameter settings are unlikely to perfectly label all the seeding sites in such a complex dataset. Therefore, the user has to select the parameters that label the seeding sites as accurately as possible and manually perform necessary corrections. Figure 8A (lower panel, t^0^) displays the same seeding site (yellow arrowhead) after the correction (seeding site splitting). Other types of mistakes introduced by the segmentation algorithm are under-segmentation and the labelling of noise without biological significance.

### Comparison of the workflows

The outcome of the 3D approaches is compared to the 2D manual approach, as it has been considered a state of the art in the field and was utilised in various previous publications [2, 27, 28, 30, 37]. When developing the 3D workflow, we did not attempt to evaluate the new model on the outcome of the 2D method, that is, our goal was not to achieve the same results as with the 2D method. Instead, we replaced all parts of the 2D workflow by suitable 3D substitutes with the goal to automate the method as far as possible in order to achieve a substantial time saving. We improved our 3D workflow until we found the results were good enough by visual inspection. Only then we compared the 3D approaches to the 2D approach and observed that we obtain very similar results while being much faster and capturing information about the 3D objects that previously were unavailable.

The manual correction of ROI identification and matching are the most time-consuming parts of the 2D workflow, and it takes on average 100 min for the complete analysis (Fig. 9B). In case of using the 3D semi-automatic approach, this time is reduced by approximately 70% to less than 30 min. The most time-consuming part in this case are the manual corrections of the segmentation, the corrections of incorrectly matched seeding sites, and the manual tuning of the processing parameters. Manual corrections however, on average, are required for approximately 7% of the labels. Most often the labels have to be manually merged or split, followed by reassigning wrongfully matched label IDs. Deleting falsely detected labels needs to be done rarely, for ca. 2% of labels. Lastly, when using the 3D automatic approach, this time is further reduced to approximately 15 min, needed only for manual tuning of the parameters. Processing time of automatic parts of the workflow is approximately 3 min. We propose using the 3D semi-automatic workflow for smaller datasets where time constraints are less critical, and the 3D automatic workflow for larger datasets, as it remains reliable and offers significant time savings.

Utilising the 3D approach enables an easier automatic segmentation as the additional dimension ensures the spacing between the seeding sites, which allows for a better distinction between them as compared to the 2D projection approach. Therefore, most of the workflow can be automated, resulting in a substantially faster analysis. Besides the significant time savings, both 3D workflows offer a more sophisticated analysis of NMJs for each individual time point, providing information about morphological properties as well as information about intensities for each individual NMJ.

### 3D workflow limitations

The user has to be careful when deploying the proposed workflow and should bear its limitations in mind. Some of them are discussed below.

For the datasets analysed within this study, linear registration was used. It performs satisfactory, aligning the majority of the objects accurately. However, sometimes small objects do not get aligned. It is then up to the user to decide whether (a) the objects are actually the same seeding site and should be matched (the user manually matches the objects) or (b) the objects are small and appear physically close but are not actually the same seeding site (no manual matching is required then). Non-linear registration could possibly be used for matching the small objects (case (a)) as it allows for localised deformation within the images (frameworks like *elastix* or *ANTs* may serve for elastic registration [18, 45]). From the authors’ experience, however, it is easier to match the seeding sites manually rather than to compare images with local deformations and assess whether the matching of seeing sites is justified. The choice of registration algorithm therefore requires a fine balance between mismatching the small objects using linear registration and wrongfully matching small objects via elastic registration. Furthermore, elastic registration of small objects is generally difficult, as they have a small weight when computing the registration metric and, hence, even elastic registration may not be able to overcome the missed matchings of small objects.

Images acquired by confocal microscopy often exhibit anisotropic voxel size in the third dimension (Z axis), resulting in a smear of an imaged object along that axis. This influences the segmentation and the objects in images with lower Z axis resolution may appear larger than the segmented objects in images with higher Z axis resolution. Calculating the overlaps of objects in images with different Z axis resolution may therefore lead to a bias. However, as the images within our study have a constant voxel size, this does not pose an issue. We recommend the potential users to use datasets acquired with the same voxel size, particularly along the Z axis, to avoid biases in segmentation, object size estimation, and overlap calculations.

As previously mentioned, manually correcting the intermediate results might result in user bias. To minimise the error due to the user’s subjectivity, proofreading and error correction should be done by the same person within a study.

## Conclusion

We here introduce a novel and fast 3D in-vivo imaging analysis workflow. We developed this workflow for confocal image analysis over distinct time points to analyse the dynamics of the EGFP-tagged seeding protein Liprin-*α*. However, as the workflow is quite general, it should be applicable to other pre- and postsynaptic events, e.g. visualising Bruchpilot or glutamate receptors dynamics. Following the changes in single NMJs over multiple time points would also be possible. Due to the reduced work time and increased accuracy of the 3D semi-automated workflow, such a holistic analysis has become feasible and might yield substantial insights into the molecular pathways underlying the process from stochastic seeding to mature and stable distribution patterns of synapses at the synaptic terminal. With the help of our fast and accurate 3D analysis, we have opened the road to a quantitative, comparable and complex multi-protein analysis of the fundamental process of synaptogenesis underlying the general principles of locomotion, learning and memory.

## Availability and requirements

*Project name*: –

*Project home page*: –

*Operating system(s)*: Platform independent

*Programming language*: C++ and Python

*Other requirements*: Development version of Amira software

*License*: MIT-style

*Any restrictions to use by non-academics*: None

## Figures and Tables

**Fig. 11:**
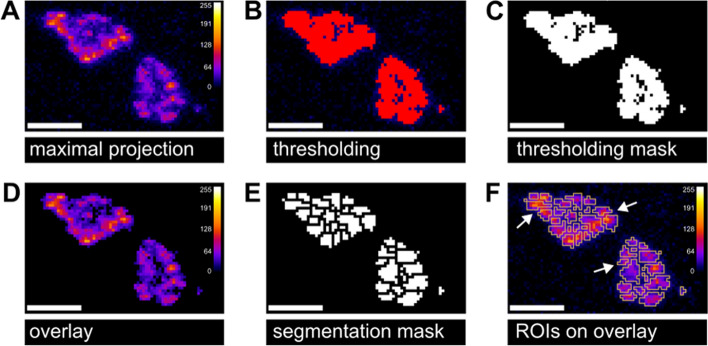
Procedure of the 2D manual workflow. **A** A maximal projection is generated from the normalised 3D stack depicting two boutons of an NMJ expressing Liprin-*α*^EGFP^. **B** Thresholding should exclude as much irrelevant signal as possible while maintaining NMJ integrity, **C** resulting in a black and white mask. **D** The thresholding mask is then subtracted from the maximal projection resulting in an overlay. **E** Maxima detection is used to automatically segment the image leading to a mask from which **F** ROIs are automatically detected. The ROIs are then layed over the maximal projection (**A**). In many cases manual refinement of the ROIs is necessary (white arrows). The colour scale indicates the pixel intensity value. Scale bar 2 μm

## Data Availability

The software can be downloaded from https://amira.zib.de/download.html. The data can be obtained at https://www.zib.de/ext-data/2026_Brence_et_al_Supplementary-Material.zip.

## Appendices

### Appendix 1: Implementation details

**Normalisation implementation**

The following operations were used to realise the preprocessing of 8-bit Z-stack images (*image1*) from original microscopy files (path to the plug-in within *FIJI* and parameter values are specified) (Fig. 10):

Maximum intensity projection—*Image/Stacks/Z Projection*:

- Working image: *image1* (8-bit Z-stack image)
- Start slice: 1
- Stop slice: Image-dependent (last slice of the image)
- Projection type: Max Intensity

Measure background—*Analyse/Measure*:

- Measure background mean intensity value from three representative areas of *image1* maximal intensity projection

Background subtraction—*Process/Math/Subtract*:

- Working image: *image1*
- Subtract: Background mean intensity value
- Save as *image2*

Intensity distribution restoration—*Process/Math/Multiply*:

- Working image: *image2*
- Multiply: 255/(255 − n), n being background mean intensity value
- Save as *image3*

**2D workflow implementation**

The 2D workflow implementation was adapted from [5] utilising existing plugins within the open-source programme FIJI, version 1.54j, 12 June 2024 [36]. The workflow consists of maximal intensity projection, thresholding, automatic segmentation, automatic ROI selection and manual refinement of ROI selection (see Fig. 11).

The following operations were taken in the listed order to realise the 2D workflow (path to the plugin within *FIJI* and parameter values are specified):

Maximum intensity projection—*Image/Stacks/Z Projection*:

- Working image: *image3*
- Start slice: 1
- Stop slice: Image-dependent (last slice of the image)
- Projection type: Max Intensity
- Save as *image4*

Thresholding—*Image/Adjust/Threshold*:

- Working image: *image4*
- Lower threshold: Image-dependent
- Upper threshold: 255
- Method: Default
- Dark background: Checked
- Do not reset range: Checked
- Save as *mask1*

Minimum overlay generation—*Process/Image Calculator*:

- Image1: *image4*
- Operation: Min
- Image2: *mask1*
- Create new window: Checked
- Save as *image5*

Second thresholding—*Image/Adjust/Threshold*:

- Working image: *image5*
- Lower threshold: 1
- Upper threshold: 255
- Method: Default
- Do not reset range: Checked

Maxima detection—*Process/Find Maxima*:

- Image1: *image5*
- Prominence: Dataset-dependent (5.00 was used within the study)
- Exclude edge maxima: Checked
- Above lower threshold: Checked
- Output type: Segmented particles
- Save as *mask2*

Second minimum overlay generation—*Process/Image Calculator*:

- Image1: *mask2*
- Operation: Min
- Image2: *image4*
- Create new window: Checked
- Save as *image6*

Third thresholding—*Image/Adjust/Threshold*:

- Working image: *image6*
- Lower threshold: Dataset-dependent
- Upper threshold: 255
- Method: Default
- Dark background enabled: Checked
- Do not reset range: Checked

Single Liprin-*α* seeding sites analysis—*Analyze/Analyze Particles*:

- Working image: *image6*
- Size: 2-Infinity
- Pixel units: Checked
- Circularity: 0.00–1.00
- Add to Manager: Checked
- Exclude on edges: Checked

Seeding site refinement—*Analyze/Tools/ROI Manager*:

- Working image: *image6* of each time point
- Manual refinement of ROIs is obtained using *Polygon selections*
- For individual identification, each ROI is compared in the processed images of the different time points and corrected accordingly
- Finally, ROIs are named individually and counted
- Save ROI set

Seeding site analysis—*Analyze/Tools/ROI Manager*:

- To extract information about single ROIs (i.e. Liprin-*α* seeding sites), the *Measure* option can be used (e.g. size or mean pixel intensity).

**3D workflow implementation**

The 3D workflow was implemented within Amira [40], since many workflow steps could be directly executed using existing tools for image processing and visualisation, including modules for image filtering, registration, segmentation, 2D/3D visualisations, and interactive data editing.

The following operations were used to realise the 3D workflow (module names in Amira and parameter values are specified):

Filtering—*Non-local means filter*:

- Data: *image3*
- Mode: GPU Adaptive Manifold
- Interpretation: 3D
- Spatial standard deviation: 5
- Intensity standard deviation: 0.2
- Search window: 10
- Local neighborhood: 3

Segmentation—*Hierarchical Watershed Segmentation*:

- Data: Filtered image
- Interpretation: 3D
- Neighborhood: 26
- Watershed mode: Bright
- Global range: Dataset-dependent
- Filter range: Dataset-dependent
- Detail level: Dataset-dependent

Segmentation correction—*Coral Segmenter*: The user has to interactively draw scribbles over the labels that need to be corrected (Fig. 12). The following options are available:

- Option *cut* to split a label
- Option *merge* to join different labels
- Option *assign* (to 0) to remove a label

Image duplication—*Arithmetic*: The module is used to duplicate image objects to be used for registration. The module is used twice.

- Input A: Filtered intensity image at t^i^ or segmented image at t^i^
- Result type: input A
- Expression: a

Registration—*Register Images*:

- Model: Duplicated filtered intensity image at t^i^
- Reference: Filtered intensity image at t^i+1^
- Transform: Dataset-dependent (within this this study we used Rigid, Iso-Scale, Aniso-Scale, Shear)
- Register: 2D or 3D
- Metric: Normalized Mutual Information
- Optimizer step: Default

Transform copying—*Copy Transformation*: The module is used to copy the transformation information from registered filtered image to the duplicated segmented image.

- Data: Duplicated segmented image
- Reference: Duplicated registered filtered image

Resampling—*Resample Transformed Image*: This module resamples the image to the coordinate system of the reference image, utilising linear interpolation.

- Data: Registered filtered intensity image at t^i^
- Reference: Filtered intensity image at t^i+1^

Label Matching—*Relabel Cell IDs*:

- Data: Registered and resampled segmented image at t^i^
- Label field: Labelled image at t^i+1^

Seeding site analysis (comparison of labels between time points)—*Compute Label Difference*: This module provides information about which labels have been matched, which have disappeared and which have newly appeared. It also provides the overlap and relative overlap voxel counts for each matched segment.

- Reference field: Registered and resampled segmented image at t^i^
- Data field: Segmented image at t^i+1^

Seeding site analysis (analysis of labels in individual time points)—*CalculateLabelFieldStats*: Module is used to get information about Liprin-*α * seeding site diameter, raw integrated density and mean intensity.

- Label field: Non-registered segmented image with matched labels
- Image: Registered intensity image
- Save directory: Path to the directory where the user wants results saved

Seeding site analysis (analysis of individual time points)—*Label Analysis*: Module is used to get information about anisotropy, elongation and flatness.

- Data: Non-registered segmented image with matched labels
- Interpretation: 3D
- Measures: Standard Shape Analysis

Visualisation—*Ortho Slice* and *Color Wash*:

*Ortho Slice* can be used to visualise any dataset as orthogonal 2D slices. The user can additionally attach the *Color Wash* module and overlay the displayed dataset with another dataset. The user can scroll through the slices.

Visualisation—*Voxelized Rendering*:

Module *Voxelized Rendering* can display any image in 3D. The user can view the entire dataset and view it from an arbitrary angle. Different colour maps can be selected to easily discriminate between the labels (seeding sites).

### Appendix 2: Graphical flowchart of the 3D workflow

This section provides a more detailed flowchart of the 3D workflow (Fig. 13). Compared to Fig. 4, it shows each processing step, analogous to the “Processing workflows” section 3D workflow.

## Abbreviations

2D: Two dimensions or two-dimensional
3D: Three dimensions or three-dimensional
BRP: Bruchpilot
e.g.: Exempli gratia
EGFP: Enhanced green fluorescent protein
ID: Identifier
i.e.: Id est
LLPS: Liquid–liquid phase separation
N.A.: Numerical aperture
NMI: Normalised mutual information
NLG-1: Neuroligin-1
NLM: Non-local means
NMJ: Neuromuscular junction
NRX-1: Neurexin-1
ROI: Region of interest
SYD-1: Synapse-defective 1
SYD-2: Synapse-defective 2
UNC13: Uncoordinated-13
VGCCs: Voltage-gated calcium (Ca^2+^) channels
w.r.t.: With respect to
